# Resolving early obesity leads to a cardiometabolic profile within normal ranges at 23 years old in a two-decade prospective follow-up study

**DOI:** 10.1038/s41598-021-97683-9

**Published:** 2021-09-23

**Authors:** Paulina Correa-Burrows, José Rogan, Estela Blanco, Patricia East, Betsy Lozoff, Sheila Gahagan, Raquel Burrows

**Affiliations:** 1 Instituto de Nutrición y Tecnología de los Alimentos, Universidad de Chile, Avda. El Líbano 5524, Macul, CP: 7830490 Santiago, Chile; 2 Departamento de Física, Facultad de Ciencias, Universidad de Chile, Santiago, Chile; 3 Centro Para la Nanociencia y la Nanotecnología, CEDENNA, Santiago, Chile; 4 Division of Child Development and Community Health, University of California San Diego, La Jolla, CA USA; 5 Center for Human Growth and Development, University of Michigan, Ann Arbor, MI USA

**Keywords:** Risk factors, Biomarkers, Epidemiology, Paediatric research, Predictive markers

## Abstract

Obesity is the most important predisposing factor for cardiovascular disease and type-2 diabetes. We explored the relationship between the age at onset of obesity and selected cardiometabolic parameters in young adults. Longitudinal study of *n* = 1,039 participants (48% males) in their early twenties. BMI was measured at birth, 1–5–10–12–14–16–23 years. BMI trajectories were interpolated. Five groups were identified: never obese (never-OB); early childhood obesity transitioning to non-obesity before adolescence (former-OB); obesity starting in preadolescence transitioning to non-obesity as adolescents (transient-OB); obesity from adolescence into early adulthood (recent-onset-OB); participants who were obese in early childhood and remained obese into adulthood (persistent-OB). Waist circumference (WC), blood pressure, lipids, glucose, and insulin were measured at 23 years. HOMA-IR and the Metabolic Syndrome Risk Z-Score were estimated. In the sample, 47% were obese during at least one time-point. Mean obesity duration was 20.7 years, 8.5 years, 6.2 years, and 3.3 years in persistent-OBs, recent-onset-OBs, former-OBs, and transient-OBs, respectively. The cardiometabolic profile was more adverse in recent-onset-OBs (12%) and persistent-OBs (15%) compared to never-OB participants (53%). Although former-OBs (15%) and transient-OBs (4%) had higher WC values than never-OBs, no differences were seen in other biomarkers. Both persistent and recent-onset obesity led to a cardiometabolic profile of risk in early adulthood, as suggested by values of WC, HOMA-IR, and hs-CRP above normal limits and HDL-chol values below normal limits. Participants who had obesity in early childhood or preadolescence but transitioned to a non-obesity status had a cardiometabolic profile similar to participants who were never obese and within normal limits. Obesity leads to risky values in a number of cardiometabolic biomarkers in young adulthood independent of age at obesity onset. Likewise, overcoming obesity during the pediatric age leads to a cardiometabolic profile within normal ranges at 23 years of age.

## Introduction

The obesity epidemic places a heavy burden on individuals, societies, and the economies in the form of multiple chronic diseases (e.g., cardiovascular, liver and kidney diseases, and type-2 diabetes), reduced life expectancy and quality of life, and substantial pressure on health budgets^1–3^. In Chile, the prevalence of obesity increased remarkably in the last three decades^4–7^. In the mid-1980s, less than 5% of schoolchildren had obesity; in 2019, 25% were 2 *SD* above WHO Child Growth Standards median for age and sex. According to the last National Health Survey, 74% of Chilean adults are either overweight or obese^5–7^.

Many longitudinal studies conclude that childhood obesity relates to a higher risk of developing cardiometabolic diseases in adulthood due to obesity persistence over time^8–10^. The early timing of adiposity rebound or the second rise in BMI following a nadir in early childhood has been associated with increased risks of persistent obesity and metabolic imbalances in adolescence and adulthood^11–13^. Adiposity rebound should occur at 5–6 years, but this increase in BMI happens much earlier in most children. Because children with early obesity are generally accepted to be at a high risk of adverse cardiometabolic profiles, identifying these individuals at risk timely to allow possible interventions and support is a major challenge in most countries where childhood obesity keeps rising. From a scientific standpoint, however, the role of age at obesity onset in subsequent cardiometabolic risk remains a controversial topic. While several studies show a relationship between obesity in early childhood and cardiometabolic risk later in life^11,14–20^, others suggest that the risk of experiencing vascular events or developing type-2 diabetes () in adulthood relates to the magnitude and/or duration of obesity, independent of age at obesity onset^21–24^. Most studies longitudinally exploring this relationship use a retrospective approach to the problem by going back in time to identify groups of exposed or unexposed subjects and compare the incidence of cardiometabolic diseases. Although this approach may have reasonable methodological validity, it does not allow detecting findings beyond the exposed vs. unexposed logic; for example, what happens to the cardiometabolic profile of those temporarily exposed to the risk factor (early obesity). Aiming to expand the analysis and open up new avenues for discussion, we explore the relationship between the age at obesity onset and selected cardiometabolic parameters in young adults. To do so, we used mathematical modeling that allows prospective evaluation of each participant in the same time frame and, thus, allows more accurate analysis of BMI data as well as the role of age of onset and length of obesity in the development of cardiometabolic risk later in life.

## Methods

### Study design and population

Data for this study come from a prospective infancy cohort (the Santiago Longitudinal Study, SLS) of ≈ 1000 Chileans, 50% females, who participated in research related to nutrition and development as infants with follow-up at 1 years, 5 years, 10 years, 12 years, 14 years, 16 years, 21 years, and 23 years^21,25–29^. They were born in 1992–1996, at term, of uncomplicated vaginal births, weighed > 3.0 kg, and were free of acute or chronic health problems^25^. Enrollment occurred just as the country entered a rapid socioeconomic change associated with decreasing undernutrition and infectious disease and increasing NCD^30,31^. In 2009, when the participants were 16–17 years, they were invited to get involved in a study of biopsychosocial determinants of adolescent obesity and cardiovascular risk. Assessment of anthropometric and cardiometabolic markers was repeated at 23 years. Field-study for the 23 years old wave was conducted in 2015–2018, assessing a total of n = 1039 participants^29^. It is worth mentioning that SLS participants were born to parents or grandparents exposed to fetal and/or childhood undernutrition. This can result in developmental adaptations that produce permanent structural, physiological, and metabolic changes, predisposing an individual and the offspring to cardiovascular, metabolic, and endocrine disease in later life^32^.

### Measurements

#### Anthropometric assessment

At 1 years, 5 years, 10 years, 12 years, 14 years, 16 years, 21 years and 23 years, research staff used standardized procedures to measure weight (kg) to the nearest 0.1 kg, using a scale (Seca 725 and 703, Seca GmbH & co. Hamburg, Germany) and height (cm) to the nearest 0.1 cm, using a Holtain stadiometer. At 23 years, waist circumference (WC) was measured in the horizontal plane midway between the lowest ribs and the iliac crest with a non-elastic flexible tape and recorded to 0.1 cm (Seca 201, Seca GmbH & co. Hamburg, Germany). Measurements were taken twice, with a third measurement if the difference between the first two exceeded 0.3 kg for weight, 0.5 cm for height, and 1.0 cm for WC^26–29^. We estimated BMI-for age (BMIz) according to the 2006 (ages 0–5) and 2007 WHO (ages 5–19) growth standards^33,34^. While the WHO weight-for-height chart is usually recommended over BMI-for-age for clinical use before the age of two, Furlong et al*.* found that the correlation between weight-for-length and BMI-for-age was very strong (r = 0.99)^35^. Similarly, we used the WHO age-19 BMI-for-age reference for BMI measured at 21 years and 23 years. By using BMI-for age in all ages, we obtained a consistent metric of relative weight across developmental stages to be analyzed as a continuous variable. Obesity was diagnosed in all participants with BMIz 2 *SD* above WHO Child Growth Standards median for age and sex. Early obesity was defined as having a BMIz ≥ 2 *SD* before the age of 6 years.

#### Cardiometabolic risk assessment at 23 years

Systolic and diastolic blood pressures (SBP and DBP) were measured three times after 15 min at rest, using a mercury sphygmomanometer, according to the 2015 U.S. Preventive Services Task Force recommendation statement; average values were used for analyses. After 8–12 h overnight fast, total serum glucose, insulin, total cholesterol, triglycerides (TG), high-density lipoprotein cholesterol (HDL-chol), high-sensitivity C-reactive protein (hs-CRP) and adiponectin were measured. Glucose was measured with an enzymatic colorimetric test (QCA S.A., Amposta, Spain), and radioimmunoassay (Diagnostic Products Corporation, Los Angeles, CA) was used for insulin determination. Cholesterol profile was determined by dry analytical methodology (Vitros; Ortho Clinical Diagnostics Inc., Raritan, NJ). Serum hs-CRP was measured with a sensitive latex-based immunoassay, and values of > 1.0 mg/l were considered low-grade systemic inflammation, according to the AHA/CDC Joint Statement on Markers of Inflammation and Cardiovascular Disease^36^. Participants having hs-CRP values ≥ 9 mg/dl were excluded from the analysis (*n* = 171). Total adiponectin was determined with the Quantikine Human Immunoassay. The homeostatic model assessment (HOMA-IR) quantified insulin sensitivity, with values ≥ 2.6 denoting insulin resistance (IR)^37^. Metabolic Syndrome (MetS) was diagnosed based on the 2009 AHA/NHLBI/IDF Joint Interim Statement^38^. From these measures, a continuous cardiometabolic risk score was computed, according to Gurka et al.^39^.

#### Modelling of BMI trajectory

**Figure 1:**
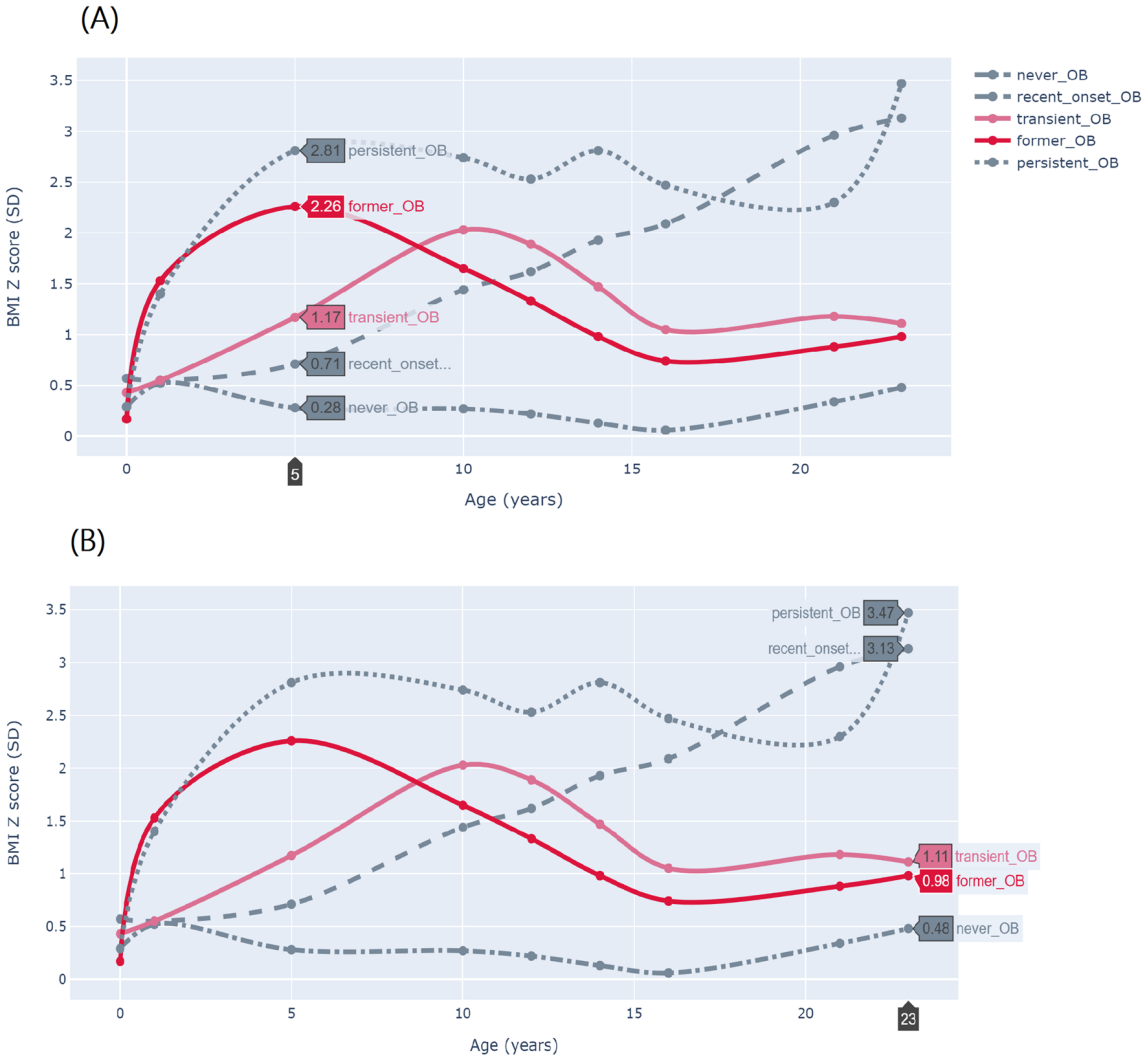
BMI trajectory from birth to adulthood in the Santiago Longitudinal Study (n = 1039). Participants who were never obese (never-OBs); participants with obesity starting in adolescence and remained obese into adulthood (recent-onset-OBs); participants who were obese in early childhood but transitioned to non-obesity as preadolescents (former-OBs); participants who were obese in early childhood and remained obese into adulthood (persistent-OBs); and participants with obesity starting in preadolescence and transitioned to non-obesity as adolescents (transient-OBs). BMI was measured at birth, 1 years, 5 years, 10 years, 12 years, 14 years, 16 years and 23 years. Trajectories were interpolated using cubic spline. This method gives an interpolating polynomial that is smoother and has smaller error than other interpolating polynomials (e.g., Lagrange and Newton polynomials). Panel (**A**) draws attention to BMI-for-age-and-sex at 5y, whereas panel (**B**) does at 23 years. Trajectories of participants that solved obesity are shown in pink (transient-OBs) and red (former-OBs) shades. Note: This figure was partially taken from Burrows et al.^42^.

We used a cubic polynomial spline to interpolate BMI trajectory from birth to early adulthood in each participant. With this method, the data points are taken from original measurements, and splines join the data points together smoothly. Spline interpolation is often preferred over other polynomial interpolation approaches (e.g., Lagrange and Newton polynomials) because it can be applied to both series of segments of the data record and entire data series, and the interpolation error can be made small even when using low degree polynomials for the spline^40^. The second advantage of this method is that it allows the construction of a smooth and visually pleasing parametric curve when dealing with sparse data^40^, particularly if the spline departs from the original data points^41^, as was the case here (we had data for birthweight in all participants). Thus, spline interpolation is more consistent whit how BMI changes over time compared to linear approaches, in which two data points are connected through a straight line. Linear interpolation uses a linear function for each interval, and although quick and easy, it is not very precise. On the other hand, Spline interpolation uses low-degree polynomials in each of the intervals and chooses the polynomial pieces such that they fit smoothly together^40,41^. As described in detail in Burrows et al*.*, we fitted models with data measured at birth, 1 years, 5 years, 10 years, 12 years, 14 years, 16 years, 21 years, 23 years, and obtained 1039 BMI trajectories from birth to early adulthood^21^. Using the full BMI trajectories available, we estimated the timing of obesity onset and duration of obesity in those participants who were ever obese or who had obesity in early childhood, with a precision of weeks. Based on observation of each interpolated BMI trajectory, we created five groups: never obese (never-OB); early childhood obesity transitioning to non-obesity before adolescence (former-OB); obesity starting in preadolescence transitioning to non-obesity as adolescents (transient-OB); obesity from adolescence into early adulthood (recent-onset-OB); participants who were obese in early childhood and remained obese into adulthood (persistent-OB) (Fig. 1; this figure was partially taken from our group’s previous work. See Burrows et al.^42^. Python 3.0 was used for data interpolation and BMI trajectory modeling.

### Data analysis

Before conducting the main analyses, we used the Shapiro–Wilk test to examine if all variables were normally distributed. The following variables were log-transformed for analysis: waist circumferences, systolic and diastolic blood pressure, HOMA-IR, insulin, adiponectin, triglycerides, and HDL-cholesterol. Non-transformed data are presented here for ease of interpretation. One-way ANOVA, the Kruskal–Wallis H test, and the Pearson χ^2^ test for independence were used for comparison of means, medians, and relative frequencies, respectively. To determine whether different BMI trajectories led to different cardiometabolic risk profiles in adulthood, we conducted an analysis of covariance with WC, SBP, DBP, FBG, insulin, HOMA-IR, TG, HDL-chol, hs-CRP, adiponectin and the MetS Risk Z-Score being the dependent variables and the obesity status according to life course BMI trajectory as the independent variable. A first model was unadjusted, and a second model controlled sex and having insulin resistance at 23 years. When insulin or HOMA-IR were the outcome variable, we adjusted for having the Metabolic Syndrome at 23 years. Post hoc analyses were done with Bonferroni adjustment to assess further differences between groups. Because independent variables were categorical, we checked the collinearity assumption after running the models. The following would denote collinearity in model with categorical predictors: very high standard errors for regression coefficients, the significance of the overall model but none of the coefficients, and large changes in coefficients when adding predictors. The results indicated that the collinearity assumption was not violated in our models. A *P* value < 0.05 denoted statistical significance. Data were analyzed using Stata for Windows version 15.0 (StataCorp. 2017. Stata Statistical Software: Release 15. College Station, TX: StataCorp LLC).

### Ethical approval

IRBs of the Institute of Nutrition and Food Technology (University of Chile), the University of Michigan, and the University of California, San Diego granted ethical approval for the study. Written informed consent was obtained from all participants and their legal guardians in assessment waves from infancy to adolescence and all participants only in the 23 years-old assessments wave, in accordance with the Declaration of Helsinki.

## Results

The mean age of participants in the early adulthood assessment wave was 23.0y (0.8 *SD*). Forty-eight percent were males, and 24.7% had obesity or a BMI above 30 kg/m^2^. According to life course BMI trajectory, 53.1% of participants were never-OB, 12.4% were recent-onset-OB, 15.0% were former-OB, 4.3% were transient OB, and 15.2% were persistent-OB (Fig. 1). Estimated mean obesity duration ranged from 20.7 years in persistent-OBs to 3.3 years in transient-OBs. The estimated mean age at obesity onset was 1.9 years, 2.3 years, 9.4 years and 14.6 years in former-OBs, persistent-OBs, transient-OBs, and recent-onset-OBs, respectively. Transition to non-obesity was estimated to take place at 8.1 years in former-OBs and 12 years in transient-OBs (Table 1). It is worth noting that a higher proportion of females was found among recent-onset OBs, whereas a higher proportion of males was found among former-OBs and transient-OBs.

**Table 1:** Anthropometric and cardiometabolic profile in 23-years-old participants in the SLS, by obesity status (n = 1039).

	Never obese (never-OBs) (n = 552)		Recently obese (recent-onset-OBs) (n = 129)		Formerly obese (former-OBs) (n = 155)		Persistently obese (persistent-OBs) (n = 158)		Transiently obese (transient-OBs) (n = 45)		*P* value*
	Mean or Median	(*SD*) or [IQR]	Mean or Median	(*SD*) or [IQR]	Mean or Median	(*SD*) or [IQR]	Mean or Median	(*SD*) or [IQR]	Mean or Median	(*SD*) or [IQR]	
**Chronological age**											
Age (years)	23.0	(1.0)	23.0	(1.1)	23.2	(1.1)	23.0	(1.1)	22.9	(0.8)	0.26
Sex distribution^‡^											
Males	257	51.6%	44	8.8%	90	18.1%	77	15.5%	30	6.0%	< 0.001^♣^
Females	295	54.5%	85	15.7%	65	12.0%	81	15.0%	15	2.7%	
**Anthropometric profile: early adulthood**											
Body-mass Index 23 year	23.6^a^	(3.1)	32.6^b^	(4.3)	25.3^c^	(2.7)	34.6^b^	(4.7)	26.2^c^	(3.3)	< 0.001
Body-Mass Index 23 year (*z* score)	0.48^a^	(1.0)	3.13^b^	(1.2)	0.98^c^	(0.8)	3.76^d^	(1.4)	1.18^c^	(1.0)	< 0.001
Body-Mass Index 21 year	22.6^a^	(2.2)	32.0^b^	(3.1)	24.1^c^	(2.7)	33.8^d^	(3.8)	25.4^c^	(3.1)	< 0.001
Body-Mass Index 21 year (*z* score)	0.34^a^	(0.8)	2.95^b^	(0.9)	0.88^c^	(0.7)	3.47^d^	(1.2)	1.09^c^	(0.8)	< 0.001
Waist circumference 23y (cm)	76.2^a^	(7.9)	92.8^b^	(8.6)	80.3^a^	(7.5)	98.2^b^	(10.1)	82.6^a^	(8.6)	< 0.001
**Anthropometric profile: infancy to adolescence**											
Birthweight (*z* score)	0.29	(0.8)	0.57	(0.8)	0.17	(0.7)	0.29	(0.7)	0.43	(1.0)	0.32
Body-mass Index 1 years (*z* score)	0.52^a^	(0.8)	0.55^a^	(0.6)	1.53^b^	(0.9)	1.40^b^	(0.9)	0.55^a^	(0.8)	< 0.001
Body-mass Index 5 years (*z* score)	0.28^a^	(0.8)	0.71^b^	(0.8)	2.26^c^	(0.7)	2.81^d^	(1.2)	1.17^e^	(0.5)	< 0.001
Body-mass Index 10 years (*z* score)	0.27^a^	(0.8)	1.44^b^	(0.9)	1.65^b^	(1.0)	2.74^c^	(1.0)	2.03^d^	(0.7)	< 0.001
Body-mass Index 12 years (*z* score)	0.22^a^	(0.8)	1.62^b^	(1.0)	1.33^b^	(1.1)	2.53^c^	(1.1)	1.89^b^	(0.9)	< 0.001
Body-mass Index 14 years (*z* score)	0.13^a^	(0.8)	1.93^b^	(1.0)	0.98^c^	(1.0)	2.81^d^	(1.0)	1.47^b^	(0.9)	< 0.001
Body-mass Index 16 years (*z* score)	0.06^a^	(0.8)	2.09^b^	(0.9)	0.74^c^	(0.9)	2.47^d^	(0.9)	1.05^c^	(0.8)	< 0.001
Age at obesity onset (years)	d.n.a	d.n.a	14.6^a^	(4.9)	1.9^b^	(1.5)	2.3^b^	(2.0)	9.4^c^	(3.1)	< 0.001
Obesity length (years)	d.n.a	d.n.a	8.5^a^	(4.8)	6.2^b^	(3.5)	20.7^c^	(2.3)	3.2^d^	(2.0)	< 0.001
**Cardiometabolic profile: early adulthood (23 years)**											
Systolic BP (mm Hg)	108^a^	(10)	115^b^	(11)	110^a^	(10)	118^b^	(12)	109^a^	(19)	< 0.001
Diastolic BP (mm Hg)	67^a^	(7)	72^b^	(7)	68^a^	(6)	74^b^	(8)	68^a^	(12)	< 0.001
Fasting glucose (mg/dl)	88.0^a^	[9.7]	89.4^a^	[9.9]	88.6^a^	[10.1]	91.0^b^	[9.8]	88.5^a^	[9.9]	0.001^‡^
Insulin (uUI/dl)	10.7^a^	[6.9]	18.4^b^	[15.0]	9.4^a^	[6.9]	17.6^b^	[15.2]	11.0^a^	[6.5]	< 0.001^‡^
HOMA-IR	2.34^a^	[1.5]	4.07^b^	[3.4]	2.06^a^	[1.6]	3.71^b^	[3.1]	2.27^a^	[1.4]	< 0.001^‡^
Triglycerides (mg/dl)	77.2^a^	[50.1]	92.6^b^	[71.0]	76.8^a^	[74.4]	108.1^b^	[81.7]	78.6^a^	[57.2]	< 0.001^‡^
HDL-cholesterol (mg/dl)	44.8^a^	(14.2)	41.4^b^	(12.7)	46.0^a^	(13.9)	38.0^b^	(11.3)	44.4^a^	(13.4)	< 0.001
Adiponectin (µg/ml)	5.97^a^	[5.8]	4.71^b^	[5.2]	6.40^a^	[5.4]	5.50^b^	[4.7]	4.83^a^	[4.2]	< 0.001^‡^
hs_CRP mg/l ^π^	1.25^a^	[1.5]	2.09^b^	[3.22]	1.25^a^	[1.4]	2.44^b^	[3.79]	1.22^a^	[1.1]	< 0.001^‡^
MetS risk Z-score (Gurka Score)	− 0.59^a^	(0.6)	− 0.03^b^	(0.7)	− 0.48^a^	(0.7)	0.27^c^	(0.6)	− 0.46^a^	(0.8)	< 0.001

*Analysis of Variance with Bonferroni correction, except as indicated. Sex distribution expressed as number of participants and percentage. ^‡^Kruskal–Wallis Test by ranks.^♣^Pearson’s Chi2 test for independence. Means or medians in a row without a common superscript letter differ, as analyzed by one-way ANOVA or Kruskal–Wallis test. ^π^Participants with hs_CRP > 9 (mg/l) were excluded from the analysis (n = 171). Anthropometric data and sex distribution were also reported in a previous work from our group. See Burrows et al*.*^42^.

A comparison of anthropometric features at 23 years shows that persistent-OBs and recent-onset-OBs had similar BMI (raw and standardized) and WC values (Table 1). Also, the WC values were similar among never-OBs, former-OBs, and transient-OBs. We did not observe differences in birth weight according to BMI trajectory group. At 1 years, however, former-OBs and persistent-OBs had a BMIz significantly higher compared to their peers, with mean BMIz in the overweight category (1.5 and 1.4 *SD*, respectively). At 5 years, BMIz remained higher in former-OBs and persistent-OBs compared to the other groups, with values denoting obesity; yet persistent-OBs had higher BMIz than former-OBs. At 10 years, the highest BMIz was observed in persistent-OBs followed by transient-OBs, both in the obesity range. At that point, former-OBs were no longer obese. At 12 years, transient-OBs were no longer obese, with BMIz scores similar to that of former-OBs and recent-onset-OBs, within the overweight range. At 16 years, BMIz denoted obesity in persistent-OBs and recent-onset-OBs, overweight in transient-OBs, and normal weight in both former-OBs and never-OBs. Since birth, never-OBs had maintained a BMIz in the healthy-weight range.

**Figure 2:**
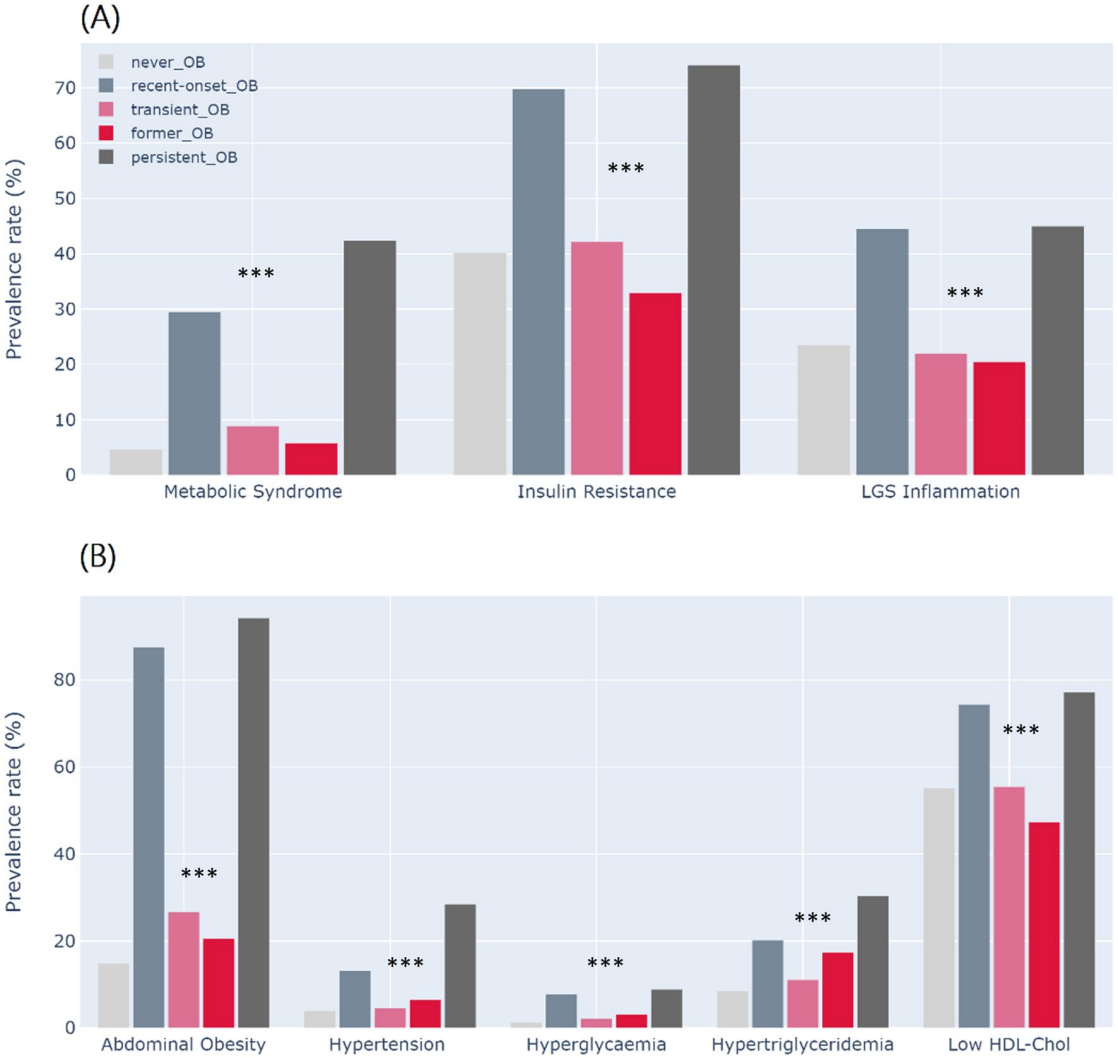
Cardiometabolic risk at 23 years by time of onset and persistence of obesity in the Santiago Longitudinal Study (n = 1039). Metabolic Syndrome (Mets) and its components were diagnosed with the AHA/NHBLI/IDF joint standard. Insulin resistance diagnosed with HOMA-IR values ≥ 2.6. Low-Grade Systemic (LGS) Inflammation diagnosed with high sensitivity C-reactive protein values ≥ 3 mg/l (participants with values ≥ 9 mg/l were excluded from de analysis; n = 171). MetS components: abdominal obesity (WC ≥ 80 and 90 cm in females and males, respectively), high blood arterial pressure (SBP ≥ 130 mmHg, DBP ≥ 85 mmHg), hypertriglyceridemia (TG ≥ 150 mg/dL), low HDL (≤ 50 and ≤ 40 mg/dL in females and males, respectively), and fasting hyperglycaemia (Gli ≥ 100 mg/dl). Participants who were never obese (never-OBs); participants with obesity starting in adolescence and remained obese into adulthood (recent-onset-OBs); participants who were obese in early childhood but transitioned to non-obesity as preadolescents (former-OBs); participants who were obese in early childhood and remained obese into adulthood (persistent-OBs); and participants with obesity starting in preadolescence and transitioned to non-obesity as adolescents (transient-OBs). Prevalence rates of participants that solved obesity are shown in pink (transient-OBs) and red (former-OBs) shades. Pearson’s Chi^2^ test for independence: *P < 0.05, **P < 0.01, ***P < 0.001.

As for the cardiometabolic profile at 23 years (Table 1), we found that never-OBs, former-OBs and transient-OBs had similar values in all biomarkers studied. While the cardiometabolic profiles of the persistent-OBs and recent-onset-OBs differed only for FBG and the MetS score, they were remarkably different from the never-OBs, former-OBs and transient-OBs. Overall in the sample, there were high prevalence rates of MetS (14%), IR (48%) and low-grade systemic inflammation (28.3%), all consistent with a cardiometabolic profile of risk. As seen in Fig. 2A, the proportions were significantly greater in recent-onset-OBs and persistent-OBs compared to the other categories, particularly in the case of IR, as at least 7 in 10 participants had the condition in early adulthood. Low-grade systemic inflammation, a condition related to dyslipidemia, atherogenesis, type-2 diabetes, and systemic arterial hypertension, was above 40% in persistent-OBs and recent-onset-OBs, almost twice the prevalence in the other groups. The prevalence of MetS components was also significantly higher in persistent-OBs and recent-onset-OBs compared to the other groups, particularly the prevalence of abdominal obesity (panel B in Fig. 2).

A regression analysis examined the contribution of age of onset and duration of obesity to the cardiometabolic risk profile in adulthood (Table 2). After accounting for the effect of other influences, we found that recent-onset-OBs had significantly higher values of WC (+ 16.1 cm), SBP (+ 7 mmHg), DBP (+ 5 mmHg), insulin (+ 4.3 uUI/dl), HOMA-IR (+ 0.97) TG (+ 14.0 mg/dL), hs-CRP (+ 0.89 mg/l), and MetS zscore (+ 0.05 SD), and lower values of HDL-chol (− 2.9 mg/dl) and adiponectin (− 1.05 µg/ml) than never-OBs. A very similar pattern was observed when comparing never-OBs with persistent-OBs; in this case, persistent-OBs also had higher values of FBG (+ 1.8 mg/dl) than never-OBs. It is worth noting that differences in mean values of these biomarkers denoted biological risk in WC, HOMA-IR, HDL-chol, and hs-CRP; values in recent-onset OBs and persistent-OB were above cutoffs for diagnosis of abdominal obesity, IR, and low-grade systemic inflammation and below cutoffs for low HDL-chol diagnosis. Differences found in other biomarkers did not suggest biological risk as values in recent-onset OBs, and persistent OBs were within normal values. Also in Table 2, compared to the reference group (never-OBs), former-OBs and transient-OBs only had higher WC values (+ 3.6 cm and + 5.0 cm, respectively); despite being significant, these differences did not denote biological risk whatsoever as they fell into normal ranges. In all other biomarkers considered in the analysis, we found no differences between never-OBs with former-OBs and transient-OBs, suggesting a similar cardiometabolic profile in early adulthood.

**Table 2:** Estimated regression coefficients examining the association of obesity timing and duration with cardiometabolic profile at 23 years (n = 1039).

	Obesity in early childhood (−)			Obesity in early childhood (+)	
	Never obese (never-OBs, n = 552)	Recently obese (recent-onset-OBs, n = 129)	Transiently obese (transient-OBs, n = 45)	Formerly obese (former-OBs, n = 155)	Persistently obese (persistent-OBs, n = 158)
	Intercept (SE)	Coefficient (SE)	Coefficient (SE)	Coefficient (SE)	Coefficient (SE)
**Model 1**^**a**^					
Waist circumference (cm)	76.2*** (0.36)	16.6*** (0.83)	6.35*** (1.23)	4.07*** (0.77)	22.0*** (0.76)
Systolic blood pressure (mm Hg)	108.0*** (0.47)	7.00*** (1.04)	1.25 (1.70)	2.33 (1.01)	10.4*** (1.00)
Diastolic blood pressure (mm Hg)	67.2*** (0.30)	5.21*** (0.70)	0.47 (1.12)	1.02 (0.65)	7.14*** (0.65)
Fasting glucose (mg/dl)	87.8*** (0.41)	2.31* (0.85)	1.51 (1.11)	1.32 (0.86)	3.15*** (0.87)
Insulin (uUI/dl)	12.2*** (0.35)	7.16*** (0.80)	0.87 (1.08)	− 1.38 (0.65)	7.99*** (0.64)
HOMA-IR	2.68*** (0.06)	1.67*** (0.18)	0.37 (0.21)	− 0.29 (0.10)	1.95*** (0.16)
Triglycerides (mg/dl)	91.6*** (2.58)	19.8*** (5.91)	4.21 (9.41)	9.51 (5.50)	36.1*** (5.39)
HDL-cholesterol (mg/dl)	44.8*** (0.57)	− 3.38** (1.12)	− 0.37 (2.10)	1.21 (1.10)	− 6.76*** (1.12)
hs_C reactive protein (mg/l)	2.11*** (0.07)	1.03*** (0.18)	− 0.31 (0.26)	− 0.01 (0.18)	1.12*** (0.20)
Adiponectin (µg/ml)	6.75*** (0.13)	− 1.10*** (0.32)	− 0.58 (0.51)	0.41 (0.29)	− 1.10** (0.29)
MetS rsk Z-score (Gurka score)	− 0.59** (0.02)	0.57*** (0.04)	0.14 (0.08)	0.12 (0.04)	0.87*** (0.04)
**Model 2**^**b**^					
Waist circumference (cm)	78.0*** (0.41)	16.1*** (0.67)	5.0*** (1.02)	3.6*** (0.60)	20.5*** (0.62)
Systolic blood pressure (mm Hg)	111.0 *** (0.61)	7.2*** (0.95)	− 0.2 (1.13)	1.7 (0.86)	9.5*** (0.87)
Diastolic blood pressure (mm Hg)	69.0*** (0.34)	5.3*** (0.58)	− 0.5 (0.97)	0.6 (0.51)	6.5*** (0.54)
Fasting glucose (mg/dl)	87.6*** (0.45)	1.4 (0.74)	0.9 (1.17)	1.3 (0.66)	1.8* (0.68)
Insulin^a^ (uUI/dl)	10.5*** (0.41)	4.3*** (0.59)	0.9 (1.00)	− 1.22 (0.59)	4.4*** (0.57)
HOMA-IR^a^	2.32*** (0.10)	0.97** (0.15)	0.35 (0.23)	− 0.27 (0.11)	0.96*** (0.16)
Triglycerides (mg/dl)	84.5*** (3.49)	14.0* (4.99)	3.3 (6.34)	9.7 (4.48)	29.1*** (4.58)
HDL-cholesterol (mg/dl)	43.4*** (0.67)	− 2.91* (0.92)	0.8 (1.25)	1.6 (0.80)	− 5.3*** (0.82)
hs_C reactive protein (mg/l)^π^	1.75*** (0.09)	0.89*** (0.19)	− 0.21 (0.23)	0.07 (0.07)	1.04*** (0.10)
Adiponectin (µg/ml)	5.93*** (0.25)	− 1.05** (0.32)	0.56 (0.60)	− 0.43 (0.29)	− 1.31** (0.29)
MetS risk Z-score (Gurka score)	− 0.57*** (0.02)	0.50*** (0.05)	0.06 (0.08)	0.11 (0.06)	0.74*** (0.05)

Coefficients are the mean difference between a given category and the reference group (Intercept, never-OBs). ^a^Model 1 is non-adjusted. ^b^Model 2 adjusted for sex and IR (HOMA-IR ≥ 2.6) at 23 years, except as indicated. ^a^Models adjusted for sex and MetS status at 23 years. Statistical significance: ***P < 0.001; **P < 0.01; *P < 0.05. Obesity in early childhood defined as having a BMI-z 2 *SD* above the WHO growth standard before the age of 6 years. π Participants with hs_CRP > 9 (mg/l) were excluded from the analysis (n = 171).

**Figure 3:**
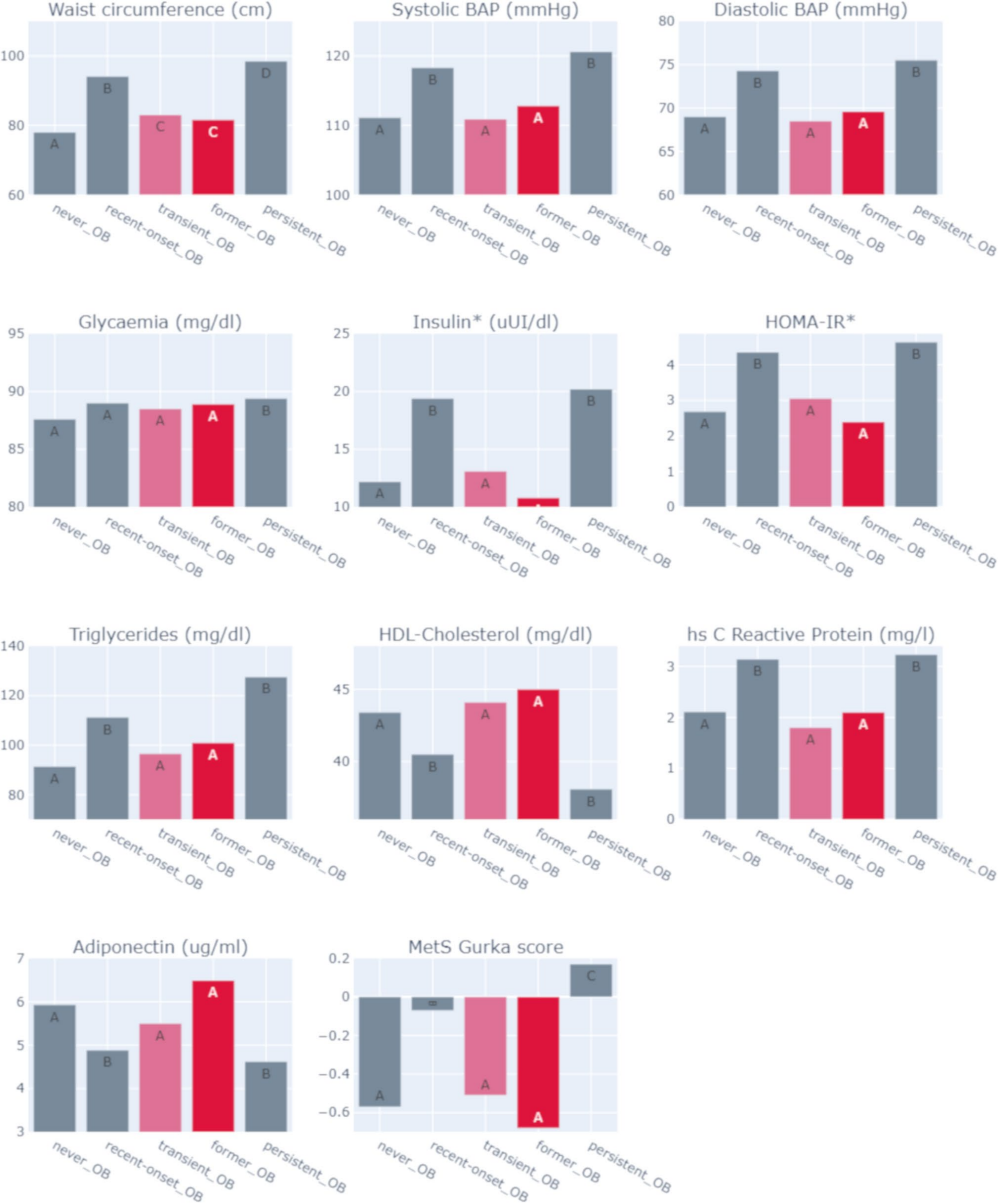
Association of age of onset and persistency of obesity with cardiometabolic profile in adulthood (23 years): ‘Post-hoc’ analysis (n = 1039). Participants who were never obese (never-OBs, A); participants with obesity starting in adolescence and remained obese into adulthood (recent-onset-OBs, B); participants who were obese in early childhood but transitioned to non-obesity as preadolescents (former-OBs, C); participants who were obese in early childhood and remained obese into adulthood (persistent-OBs, D); and participants with obesity starting in preadolescence and transitioned to non-obesity as adolescents (transient-OBs, E). Models were adjusted for sex and having insulin resistance at 23 years (HOMA-IR ≥ 2.6), except as indicated. *Models were adjusted for sex and having the Metabolic Syndrome at 23 years. Participants having hs-CRP values ≥ 9 mg/dl were excluded from the analysis (n = 171). Post hoc analysis: Letters (A–E) within a bar indicate significant differences between obesity-status categories according to Bonferroni’s test.

A *post-hoc* comparison after regression (Fig. 3), revealed the following: (1) recent-onset-OBs and persistent-OBs had similar SBP (115 vs 118 mmHg), DBP (73 vs 76 mmHg), insulin (19.4 vs 20.2 uUI/dl), HOMA-IR (4.35 vs 4.63), TG (11.4 vs 127.7 mg/dl), HDL-chol (41.4 vs 38.0 mg/dl), hs-CRP (3.14 vs. 3.23 mg/l), and adiponectin (5.65 µg/ml both groups); (2) recent-onset-OBs and persistent-OBs had increased values in all biomarkers considered in the analysis compared to former-OBs and transient-OBs; (3) persistent-OBs also had higher MetS score compared to recent-onset-OBs (− 0.07 *vs* 0.17 *SD*; *P* < 0.01).

## Discussion

### Main findings

In this study, conducted in a Chilean infancy cohort, we identified five patterns of BMI trajectories from birth to early adulthood. In previous work, in a subset of participants (*n* = 678) and using cardiometabolic outcomes in adolescence, we found four patterns of BMI trajectories^21^.

Surprisingly, we found that participants with obesity starting in adolescence (recent-onset-OBs) had a cardiometabolic profile similar to participants with persistent obesity (persistent-OBs) from early childhood. This finding confirms our results found when participants were adolescents^21^. Second, we observed that participants who had obesity in early childhood but transitioned to non-obesity in preadolescence (former-OBs) had a cardiometabolic profile within normal limits and similar to participants who never had obesity (never-OBs). Again, these findings agree with the results reported in the adolescent study^21^. Also, participants who had transient obesity and reached adulthood having non-obesity (transient-OBs) had a cardiometabolic profile similar to those who never had obesity.

Results from other longitudinal studies that track obesity status are consistent with our findings^8,22^. Results obtained in the Young Finns Study, a prospective longitudinal study initiated in the 1980s, show that long-term BMI trajectories that are persistently high or reach obesity in young adulthood are associated with cardiometabolic risk factors in adulthood. However, participants whose childhood obesity resolved show reduced risk for dyslipidemia and hypertension to the level of those who never had obesity^22^. Similarly, findings from the 1982 Pelotas (Brazil) Birth Cohort Study showed that glucose and arterial blood pressure at 30y were elevated in participants who were always overweight/obese and participants who were overweight/obese since adolescence or adulthood. Participants who were never overweight/obese or those who had overweight/obesity only in childhood or adolescence had higher HDL-chol and lower blood pressure, glucose, and LDL-chol in adulthood compared to those who were overweight/obese in adulthood^8^.

It is worth noting that different approaches used to evaluate how BMI trajectories lead to cardiometabolic outcomes may result in different findings. This might partially explain the controversy surrounding how age at obesity onset relates to subsequent cardiometabolic risk. Most studies conduct retrospective observation of longitudinal trajectories^23,24,43,44^. Participants are categorized in groups (e.g., those at high cardiometabolic risk *vs* those at low cardiometabolic risk), and then BMI trajectories are computed using past BMI data. By using this approach, researchers are constrained by the backward direction of the analysis that starts with the outcome (either a disease or risk factor) and determines the exposure. Here, we started with the exposure (BMI trend from birth onward) and, then, determined the outcome (cardiometabolic risk in adulthood).

When focusing on participants who develop obesity in early childhood (former-OBs and persistent-OBs), we observed that 50% transitioned to BMIz values < 2 *SD*, whereas the other half remained obese into adulthood. Both groups became obese around the age of 2 years. Among the former-OBs, there were more males, whereas among persistent-OBs, there were more females. Even though, both the males and the females had obesity before the age of 6 years, their cardiometabolic profiles in adulthood were different. These findings suggest that cardiometabolic risk associated with early obesity is substantially reduced when children transition to a healthy weight. In our cohort, the transition took place in preadolescence, which might be considered a window of opportunity for avoiding adverse health outcomes. Although we cannot rule out that transitions in later developmental stages have the same effect on the cardiometabolic profile, our results highlight the importance of fighting obesity during the pediatric age. Our results also suggest that persistent obesity may have a more significant impact on future cardiometabolic health than early obesity that does not persist. Since transition towards a healthy weight is vital to avoid future cardiometabolic risk, a major challenge for both health planners and clinicians is to make the process smoother. This could be particularly difficult in countries where the prevalence of childhood obesity is very high. In Chile, the prevalence of obesity in children < 6 years of age is twice that in adolescents: 27% *vs* 13%^6^. Moreover, after two years of decline (2016, 2017), the prevalence of obesity in this age-group rose again in 2018, and 2019 levels are equivalent to those of 5 years ago^6^.

Likewise, when comparing participants with transient and recent-onset obesity (transient-OBs and recent-onset-OBs), that is, participants without early obesity, we observed that transient-OBs became obese in preadolescence, whereas recent-onset-OBs became obese in adolescence. Second, transient-OBs were estimated to have in the obesity range for 3.3 years, whereas recent-onset-OBs were obese for more than eight years. In addition, females were more likely to be recent-onset-OBs and higher share of males were more likely to be transient-OBs. Again, this finding indicates that adolescence still is an opportune moment to transition back towards a healthier BMI and to avoid sustained obesity.

In our sample, persistent obesity did not lead to increased cardiometabolic risk factors at 23 years compared to recent-onset obesity. However, persistent-OBs had higher MetS score and FBG than recent-onset-OBs, and, thus, we cannot rule out that this is indicative of a higher risk of developing cardiometabolic disorders in the future.

It is worth commenting on the high prevalence of cardiometabolic risk factors among SLS participants in early adulthood. Our participants come from low SES backgrounds, and social vulnerability usually coexists with exposure to unhealthy foods, lack of exercise, and adverse psychosocial environments^45^. In most countries, the rates of income poverty tend to be low; however, multidimensional poverty, which entails deprivation in terms of health or healthy living, education, and standard of living, is much higher and may expose people to greater risk of obesity and its comorbidities^46^. Additionally, SLS participants might be expressing a thrifty phenotype resulting from being born to parents or grandparents exposed to undernutrition during fetal life. Although undernutrition is today well in the past, it was highly prevalent in Chile in the 70 s, when most of our participants’ parents were born.

Finally, a comment on the sex differences observed regarding the age of onset and duration of obesity. While a sexual dimorphism has been well-established in cardiometabolic risk, mostly related to adipose tissue distribution^47,48^, in our sample of young adults, the highest sex variations were found in the life-course trajectory but not necessarily at the level of cardiometabolic biomarkers at 23 years. In our models, sex was significant when WC, HOMA-IR, HDL-chol and adiponectin were the outcome but not in other biomarkers. As for the BMI trajectory, compared to females, males who were obese in early childhood were more likely to transition towards healthy BMI values; females who were obese in early childhood were more prone to develop sustained obesity. Likewise, males were more likely to present transient obesity. Compared to males, females were more likely to be obese starting in adolescence. These findings suggest that transitioning back to normal weight may be more difficult for females than for males and are consistent with previous work from our group. In SLS participants with diagnosis or IR, MetS or NAFLD at 23 years, we used dynamic time warping (DTW) to find optimal alignment between two time series (males vs. females), showing that males and females displayed dissimilar BMI trajectories in all cardiometabolic disorders included in the analysis, except for hypertriglyceridemia^49^. Although preliminary, these results show that the highest sex variations in the life course trajectory were observed in the case of NAFLD, hyperglycemia, hypertension, and IR. For most cardiometabolic disorders, differences in the trajectory shape were found in childhood and/or adolescence but not in early adulthood. This may partially explain why sex-related differences in cardiometabolic biomarkers at 23 years were not significant except for waist circumference, HDL-C, HOMA-IR, and adiponectin. Results reported by Schorr et al*.* in adults below the age of 40y may also help to understand ours. According to these authors, the male pattern of fat distribution was associated with a more detrimental cardiometabolic risk profile compared to women of similar age and BMI, however, visceral adipose tissue was more strongly associated with cardiometabolic risk markers in women^50^. Among SLS participants, abdominal obesity is more prevalent in females than males (43% vs 30%; *P* < 0.0001).

### Limitations and strengths

Our study has limitations that must be considered when interpreting the findings. First, we are unable to extrapolate our results to the overall Chilean population as our participants were recruited exclusively from low and middle-income families. The prevalence of obesity and obesity-related cardiometabolic risk is higher in socially vulnerable groups, according to national studies and population surveys^4,6,7^. Second, because inclusion criteria to participate in this study was a birthweight ≥ 3000 g, our cohort does not include participants with low, very low and extremely low birth weight, conditions associated with a higher risk of developing early obesity and co-morbidities later in life^51^. An additional limitation relates to the use of the WHO BMI-for-age in children < 2 years and adults > 19 years. In children 0–5 years, the standard recommends weight-for-height in clinical practice. However, Furlong et al*.*, after analyzing data for n = 1632 children aged 0–2, concluded that BMI-for-age may be an appropriate indicator of growth in the first 2 years of life with the potential to be used from birth to adulthood^35^. Likewise, aiming to address discrepancies between the child/adolescent and adult definitions of overweight and obesity in research spanning childhood and adulthood, Anderson et al.^52^ and Wright et al.^53^ used the UK age-20 BMI z-score reference for individuals 20 years of age and older. Using BMI-for-age would give clinicians and researchers the ability to use BMI from birth to adulthood and track growth trajectories using a single metric. Also, we concede that a BMIz based approach may not accurately characterize BMI levels among children with very high BMIs. While the prevalence of severe obesity among SLS participants was high at 23 years (17%), it was not that high beginning in infancy. At 1 years, less than 1% of participants (n = 8) had a BMIz ≥ 3 SD. Of them, n = 4 fall into the persistent-OB category and 4 into the former-OB category. Thus, only four individuals in the overall sample displayed a BMI trajectory of severe obesity throughout the entire life course.

Despite these limitations, the study also has several strengths. Participants were born in the early 1990s when obesity was increasing dramatically in Chile^30,31^. Because most of the participants were born to parents or grandparents exposed to childhood undernutrition, they might have inherited a ‘thrifty’ phenotype^32,54^. That is, the current sample may be representative of groups with epigenetic programming that represents a potential disadvantage for long-term health, as the ‘thrifty’ phenotype strongly relates to many chronic conditions later in life (e.g. coronary heart disease, stroke, type-2 diabetes, hypertension and certain types of cancer)^55–57^. A third strength is the availability of BMI data from birth to adulthood, with multiple evaluations across this time period. By combining both observed and interpolated data, we were able to continuously tracked participants’ growth from birth onward. This allowed longitudinal examination of trends in BMI as well as estimation of the age at which obesity starts to develop with greater precision. Additionally, BMI trends were observed prospectively, allowing identification of five patterns of growth in early childhood and subsequent obesity status and cardiometabolic risk. Most studies assessing the role of early obesity on future cardiometabolic risk rely on retrospective or backward observation of BMI trends, starting with the outcome and then looking at the exposure. Lastly, in many studies, the BMI trajectory is modelled by linear spline; here, we used polynomial functions that allow smoothing this trajectory, and thus obtain a more realistic representation of long-term BMI trends.

## Conclusion

We identified five BMI traj ectories from birth to emerging adulthood and found that both recent-onset and persistent obesity contributed to a cardiometabolic profile of risk in early adulthood—as suggested by values of WC, HOMA-IR, and hs-CRP above normal limits and HDL-chol values below normal limits—, compared to those who were never obese, formerly obese or transiently obese. Participants with sustained obesity since early childhood had higher values of a composite cardiometabolic risk score and fasting glycaemia than participants with recent-onset obesity, which could be associated with an increased risk of developing coronary heart disease and type-2 diabetes later in life. Also, we found that participants who had early-onset obesity but were able to transition to a non-obesity status as preadolescents and participants with obesity starting in preadolescence and transitioning to non-obesity as adolescents had a cardiometabolic profile in emerging adulthood similar to that of participants who never had obesity. Thus, intervening for obesity in the pediatric age, especially in children who had obesity at early ages, could be a cost-effective manner to prevent a variety of chronic diseases associated with substantial increases in disability, poor quality of life, healthcare costs and resource utilization. At this point, therapeutic interventions for treating obesity in children and adolescents might be considered in terms of investment, rather than expense.

## Data Availability

The datasets and codes generated during and/or analysed during the current study are available from the corresponding author on reasonable request.
